# The Acquisition of Directionals in Two Mayan Languages

**DOI:** 10.3389/fpsyg.2019.02442

**Published:** 2019-11-01

**Authors:** Clifton Pye, Barbara Pfeiler

**Affiliations:** ^1^Department of Linguistics, The University of Kansas, Lawrence, KS, United States; ^2^Centro Peninsular en Humanidades y Ciencias Sociales de la UNAM en Mérida, Mérida, Mexico

**Keywords:** comparative method, K’iche’, Mam, prosody, directional clitics, language acquisition

## Abstract

We use the comparative method of language acquisition research in this article to investigate children’s expression of directional clitics in two Eastern Mayan languages – K’iche’ and Mam (Pye and Pfeiler, 2014; Pye, 2017). The comparative method in historical linguistics reconstructs the grammatical antecedents of modern languages and traces the evolution of each linguistic feature (Paul, 1889; Campbell, 1998). This history informs research on language acquisition by demonstrating how phonological and morphological features interact in the evolution of new uses for common inherited traits. Children acquiring modern languages must learn the arbitrary constraints imposed on their language by its history.

## Introduction

The Eastern branch of the Mayan language family contains 13 languages including K’iche’, Mam, Ixil, Tz’utujil, Kaqchikel, and Poqomchi’. K’iche’ and Mam are spoken in western Guatemala; K’iche’ has approximately a million speakers and Mam has approximately a half-million speakers (Richards, 2003). The Eastern Mayan languages separated into the K’iche’an and Mamean subgroups more than 3000 years ago (Kaufman, 1990, 2017).

In this article, we examine the acquisition of directional clitics that K’iche’ and Mam use to express the direction an agent takes in the course of accomplishing an event. The directional clitics convey a meaning similar to the meaning that *come* expresses in the English sentence ‘We are coming to fix the sink.’ Changes to the prosody of K’iche’ and Mam triggered changes in the number of directional clitics, their position and their grammatical constraints. While Mam makes heavy use of directional clitics that precede the verb stem, K’iche’ relies more on directional clitics that follow the verb stem.

The historical changes to the common inherited trait of directional clitics show what children must learn in each language as well as alternative ways in which the directional clitics could be used. Comparisons between the directional clitics in K’iche’ and Mam increase the scope and precision of acquisition research by testing generalizations across two languages. The alternative structures of directional clitics in the other Eastern Mayan languages provide a set of potential hypotheses that children might entertain about the possible uses of the grammatical features in their own language and, thus, a better appreciation of how children acquire language-specific constraints.

We organized the presentation as follows. We begin with a description of the basic verb complex in K’iche’ and Mam. This section presents the ways in which adult K’iche’ and Mam speakers modify the verb complex to express the direction of motion. The following section provides information about the prosody of the directional clitics in K’iche’ and Mam. The section on subjects and methods provides general measures for the subjects and describes how we obtained the language data. The following section provides data on the adult and child production of intransitive directional verbs in K’iche’ and Mam. There follows a section that presents data on the adult and child production of the preverbal directional clitics in the two languages. The next section examines the adult and child production of post-verbal directional clitics in K’iche’. This is followed by a section on the production of the processive suffixes in Mam. We interpret our results in the section that applies the comparative method to the analysis of the acquisition of directional clitics in K’iche’ and Mam. The paper ends with a brief conclusion on the role of prosody in accounting for the production of directional clitics in K’iche’ and Mam, as well as in language more generally.

## The Verb Complex in K’iche’ and Mam

Robertson (1992) uses the term “the Mayan verbal complex” to refer to the combination of aspect, mood, derivational status, and cross-reference marking on Mayan verbs. The structure is syntactically complex in that it straddles the boundary between root and embedded clauses in various Mayan languages. Aspectual elements that occur in a matrix clause select indicative, nominalized and dependent types of complement clauses. The languages have a long history of pressing verbs and verb clitics into service to express aspectual, modal and directional information, and some adverbs may also be inserted into the complex (Pye, 2009).

Verb complexes in Mayan languages mark a fundamental division in transitivity. Both K’iche’ and Mam have distinct sets of absolutive and ergative person markers. The ABS person markers cross-reference the subjects of intransitive verbs and the direct objects of transitive verbs, while the ergative person markers cross-reference the subjects of transitive verbs (Pye, 2017). The verbs also have status suffixes that mark the transitivity distinction in addition to marking differences in aspect, derivational status and mood (Kaufman, 1990). The examples in (1) show intransitive and transitive indicative verb complexes in K’iche’ and Mam.


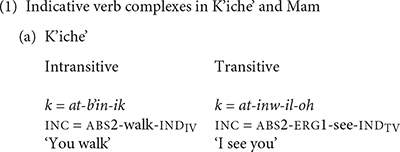



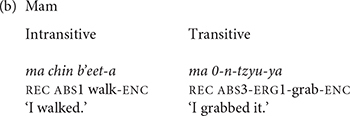


The ergative markers in both languages have different allomorphs for verbs that begin with vowels and consonants. Mam lost the second person ergative and absolutive markers and innovated an enclitic that it uses in combination with the absolutive and ergative markers to distinguish the first and second persons from the third person. The enclitic takes the form /a/ after consonants and /ya/ after vowels. K’iche’ has two personal pronouns that reference second person singular and plural in formal contexts. K’iche’ does not use the person markers on verbs with the personal pronouns. Allomorphs for the third person singular absolutive marker in Mam are a zero marker used with consonant-initial verbs, /tz’-/ used with vowel-initial verbs, /tz-/ used with the verbs *uul* ‘arrive here’ and *iky* ‘pass,’ and /k-/ used with verbs in the potential aspect. Table 1 shows the person markers for K’iche’ and Mam.

**TABLE 1:** Absolutive and ergative person markers in K’iche’ and Mam.

	**Absolutive**		**Ergative**			
		
			**Prevocalic**		**Preconsonantal**	
				
**Person**	**K’iche’**	**Mam**	**K’iche’**	**Mam**	**K’iche’**	**Mam**
1 sg	in	chin … = a	inw	w … = a	in	n … = a
2 sg	at	… = a	aw	t … = a	a	t … = a
3 sg	0	0-, tz’-, tz-, k-	r	t	u	t
1 pl exc	uj	qo … = a	q	q … = a	qa	q … = a
1 pl ixc	uj	qo	q	q	qa	q
2 pl	ix	chi … = a	iw	ky … = a	i	ky … = a
3 pl	ee	chi	k	ky	ki	ky

Mayan languages have a grammatically defined set of intransitive directional verbs. We define the directional verbs in our study grammatically rather than semantically by their incorporation into the verb complex as directional clitics in Mam. Most of the verbs in this set express the direction that the agent takes in the course of the action, although some of the verbs, like the verbs with the meanings ‘remain’ and ‘finish,’ express aspectual information. The directional verbs of K’iche’ and Mam are shown in (2).


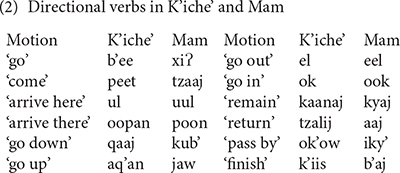


Mayan verbs lexicalize more object properties than verbs in English. Their sensitivity to object properties leads to the proliferation of Mayan verbs for eating (Brown, 2008) and breaking (Pye, 1996) among other event types. Mayan verbs of object transfer are sensitive to the way in which objects are carried. Mayan women carry small children in their arms (K’iche’ *q’aluuj*), or on their backs by means of a shawl tied around their shoulders (K’iche’ *eqaaj*). They carry water in jars balanced on top of their head (K’iche’ *ikraj*). Mayan men and women carry heavy burdens perched on their backs with a tumpline strapped across their foreheads (K’iche’ *teleej*). They carry mats over their shoulders (K’iche’ *jekeej*), and they carry smaller objects in bags at their sides or in their hands (*k’am*).

The lexicalization of object and manner properties in Mayan verbs takes priority over expression of the direction of motion. One way that K’iche’ and Mam can express the direction in which the agent travels is by adding directional clitics to the verb complex (3). The directional clitics derive historically from the directional verbs, but became grammaticalized as verb clitics. One result of grammaticalization is that the directional clitics contribute a directional component to the meaning of the verb complex rather than a motion component. The directional clitic *ul* in K’iche’ is cognate with the directional clitic *uul* in Mam, but its meaning changed to ‘come’ in K’iche’ from the original meaning of the directional verb ‘arrive here’ preserved in the Mam clitic. The entire complex has one marker for aspect and one for absolutive person marking. Verbs with directional clitics have dependent status suffixes in many contexts (Mondloch, 1978).


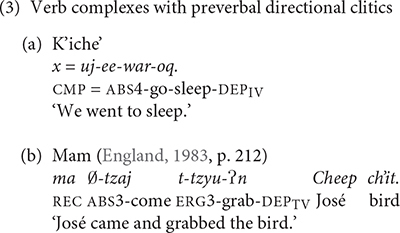


These examples show that the K’iche’ directional -*ee* ‘go’ and the Mam directional -*tzaj* ‘come’ are placed after the aspect and absolutive markers and before the verb, if intransitive (3a), or before the ergative subject marker if the verb is transitive (3b). The single ABS person marker -*uj* ‘we’ in (3a) cross-references the subject of the intransitive verb *war* ‘sleep.’ The null third person absolutive marker in (3b) cross-references the object of the transitive verb *tzyu* ‘grab.’ The directional clitics indicate the motion that the agents take in the event.

Mayan verb complexes with directional clitics are translated into English in several ways including a verb with an infinitive complement (3a) or a compound verb construction (3b). These options are also available in K’iche’ and Mam, but K’iche’ and Mam speakers prefer the use of verb complexes with directional clitics. The differences between the Mayan verb complexes with directional clitics and their English translations encapsulate the basic problem that clause chains create for theories of language acquisition, i.e., events that are expressed by several clauses in some languages may be expressed by a single clause in other languages (Harris, 2003).

The examples of verb complexes with directional clitics in (3) differ from those in (1) in that the verb complexes in (3) incorporate a directional clitic, and end with a dependent status suffix rather than the indicative status suffixes shown in (1a) for K’iche’. The dependent status suffixes are evidence that the verb complexes with directional clitics derive historically from complex sentences with dependent clauses (Pye, 2009). The presence of a single marker for aspect and absolutive affixes for the verb complexes in (3) is evidence that verb complexes with directional clitics function synchronically as single verb complexes. Independent directional verbs require their own aspect and absolutive markers. The directional clitics uniformly modify the action of the agent and not the action’s effect on the patient.

Mam elaborated the expression of direction with more preverbal directional clitics than K’iche’. K’iche’ only has three preverbal directional clitics: *e* ‘away,’ *ul* ‘hither’ and *ok’ow* ‘passing.’ Mam has 12 directional clitics in preverbal position. Directional clitics are optional in K’iche’ but obligatory for all but three transitive verbs in Mam (England, 1983, p. 170). Furthermore, K’iche’ only allows the use of one directional clitic at a time, whereas Mam allows sequences of up to three directional clitics. The directional clitics *jaw* ‘up,’ *xi* ‘away’ precede the main verb *ii* ‘carry’ in the Mam verb complex shown in (4). The entire complex only has one aspect marker (*ma*) as well as a single subject (*w*-) and object marker (*0*-). The main verb has the transitive dependent suffix/-*?n*/to indicate that the verb complex contains directional clitics.


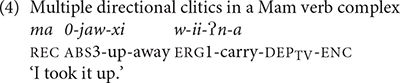


England (1983, pp 170–171) notes that each transitive verb in Mam is closely associated with its own set of directional clitics. The directional clitics also have secondary senses. For example, the directional *xi* has a primary sense of ‘away’ and a secondary sense of ‘incipience.’ The corresponding directional clitic in K’iche’ *ee* has a primary sense of ‘go’ as well as a secondary sense of ‘incipience.’ The Mam directional *el* has a primary sense of ‘out’ and a secondary sense of ‘motion to the west.’ The directional *kyaj* has a primary sense of ‘leave here’ and a secondary sense of ‘completion.’ The directionals *xi* ‘away’ and *b’aj* ‘complete’ are the most frequently cited directionals in over a third of transitive verbs in Mam, and especially with verbs that lack a directional component in their meanings. The directionals *kub’* ‘down,’ *jaw* ‘up,’ *el* ‘out’ and *ok* ‘in’ correspond to the four cardinal directions of the Mayan cosmos: down, up, west, and east.

Although the directional verbs have meanings that overlap with those of other motion verbs such as *fall* or *walk*, their incorporation in the Mam verb complex distinguishes them from all other verbs. The directional clitics follow the directional verbs in Mam but precede non-directional verbs such as *fall* or *walk*. The directionals *xi* ‘away/incipience,’ *kyaj*, ‘leave here/completion’ and *b’aj* ‘complete’ have semantic components that are aspectual as well as directional. The K’iche’ verbs *tajin* ‘continue,’ *majiij* ‘begin,’ *tanab’a’* ‘finish’ and their counterparts in Mam have aspectual meanings as well, but do not incorporate into the verb complex like directional clitics. Thus, the directional verbs form a closed, grammatical class rather than a semantic class.

The directional verb *b’ee* ‘go’ in K’iche’ has a suppletive imperative form. Imperative verbs with the directional clitic *b’ee* ‘go’ in K’iche’ use the suppletive form of the directional verb. Imperative verbs with the directional clitics *ul* ‘hither’ and *ok’ow* ‘passing’ in K’iche’ use the regular imperative prefix *ch*-. The main verb in both cases takes the dependent status suffix (5a). The directional clitics follow imperative verbs and contract in Mam (5b).


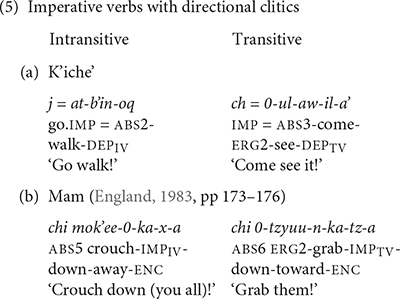


The change from preverb position to post-verb position for directional clitics on imperative verbs in Mam (in 5b) is noteworthy because change in position is one of the defining features of clitics (Zwicky and Pullum, 1983). The absence of an aspect marker to host the directional clitics on imperative verbs in Mam triggers the movement of the directional clitics to a post-verbal position. This change echoes the change in K’iche’ from preverbal clitic to post-verbal clitic and is evidence of a common underlying structure that continues to direct the historical development of directional clitics in K’iche’ and Mam.

In addition to these shared means of expressing direction, K’iche’ and Mam each have language-specific ways of expressing direction. K’iche’ has a set of post-verbal directional clitics that are used in combination with the main verb to indicate the direction of the action. The preverbal directional clitics are a subset of clitics that occur in post-verbal position (6).


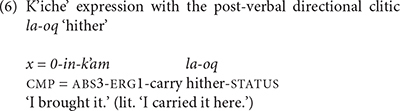


Like the preverbal directional clitics, the K’iche’ post-verbal directional clitics derive from the directional verbs. They have suffixes like the status suffixes of intransitive verbs (7). Unlike the preverbal directional clitics, the post-verbal directional clitics do not trigger the use of the dependent status suffix on the main verb. Mam does not have a separate set of post-verbal directional clitics.


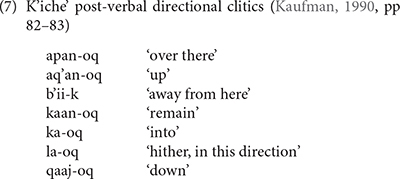


Mam has its own unique way to express direction using the processive suffix with the meaning ‘movement away.’ The processive suffix has different forms for declarative and imperative verbs (8). Unlike the directional verb clitics, the processive suffix does not trigger the use of the dependent status suffix on the main verb. K’iche’ does not have a processive suffix.


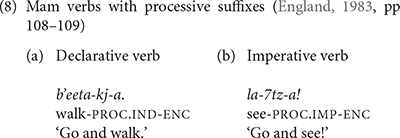


The presence of directional clitics in K’iche’ and Mam is evidence of a shared antecedent for the structures in the two languages. Mam elaborated the preverbal clitics, while K’iche’ elaborated its post-verbal clitics. Table 2 summarizes the features of directional clitics in K’iche’ and Mam. We present the morpheme frequency data for K’iche’ and Mam in the results section of the paper.

**TABLE 2:** Features of directional expression in K’iche’ and Mam.

**Feature**	**K’iche’**	**Mam**
Directional verbs	Frequent	Frequent
Preverbal directional clitics	Rare	Almost obligatory
Post-verbal directional clitics	Frequent	Only with imperative verbs
Number of preverbal directional clitics	3	12
Number of post-verbal directional clitics	7	None
Multiple directional clitics	No	Yes
Processive suffix	No	Rare

## Prosody

The structural differences in the expression of direction in K’iche’ and Mam reflect changes to the prosodic structure in the two languages. Primary lexical stress occurs on the final syllable in K’iche’ (Norman, 1976). This syllable happens to be the syllable that contains the status suffixes on verbs and post-verbal clitics. Phrasal stress shifts to the final clitic following the verb complex, in effect metrically connecting the directional clitics in K’iche’ to the verb complex. Stress in Mam is determined by syllable weight, falling on the long vowel in a word or on the vowel preceding the last glottal stop. In words that lack long vowels or glottal stops, stress occurs on the vowel preceding the last consonant in the root (England, 1983, pp 37–38). Bennett (2016) provides an overview of stress in the Mayan languages including K’iche’ and Mam.

K’iche’ and Mam also differ in the realization of unstressed vowels. They are retained in K’iche’ but deleted in some contexts in Mam (England, 1983, pp 43–44). The deletions in Mam are apparent in comparisons between cognate words in K’iche’ and Mam (9). K’iche’ retains the initial /a/ in (9a), the initial /u/ in (9b) and the second /o/ in (9c). The cognate words in Mam have one less vowel.


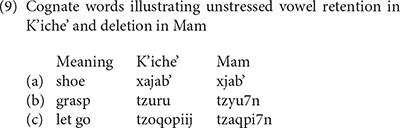


England states that the directional clitics retain their status as phonological words in that a pause can occur after them and they can also receive stress, although they are usually not stressed in connected speech (England, 1983, p. 40). Additional evidence for the phonological independence of the preverbal directional clitics, as well as the aspect and ABS person markers in Mam, is that they do not undergo the deletion of their vowels in syllables preceding the stressed vowel in a root as in the words in (9). This is the reason that Mam linguists follow England’s convention of writing the Mam verb complex with a space following the directional clitic as shown in (4).

The combining forms of the preverbal directional clitics in Mam provide additional evidence of vowel deletion in unstressed syllables. England (1983, p. 168) states that when the clitics *xi* ‘away’ and *tzaj* ‘toward’ combine with other clitics they have the reduced forms -x and -tz respectively (10). Vowel preservation in the aspect marker *ma* ‘recent past’ and the combined forms of the directional clitics shows that these morphemes retain enough phonological independence to resist stress-based vowel loss. The combining forms of the directional clitics form a separate phonological word that retains at least one vowel, but reduces the second vowel.


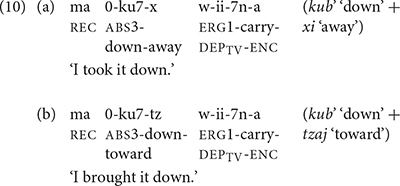


K’iche’ adds status suffixes to verbs and post-verbal directional clitics when they occur in the final position of a phonological phrase. The status suffixes delete or change form in non-final positions. The final position is also where K’iche’ adds phrasal stress. Phrasal stress in K’iche’ triggers the addition of most status suffixes. The K’iche’ examples in (11) contrast the forms of the post-verbal clitic *kan*-*oq* ‘remain’ in non-final and final positions in the verb phrase. The clitic *kan* has lexical stress in (11a) and phrasal stress in (11b).


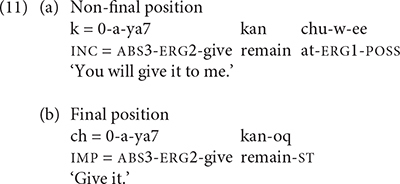


The historical relation between K’iche’ and Mam informs the structural differences between the two languages and their effects on language development. K’iche’ integrated the prefixes of its verb complex more tightly while increasing the prominence of the final syllable of the complex. The prosodic salience of syllable-final stress in K’iche’ reduced its reliance on preverbal clitics and instead promoted the prosodic salience and use of the post-verbal clitics. In effect, the change to final syllable stress in K’iche’ shifted the expression of direction from a weak syllable to a strong syllable by the change from preverbal directional clitic to post-verbal directional clitic. The number of preverbal clitics became reduced, while the number of post-verbal clitics increased in order to maintain a means of expressing direction for verbs that lack a directional component in their meaning. Romero (2012) shows that negation particles underwent a similar change in K’iche’.

The pieces of the verb complex became less metrically integrated in Mam with the result that the directional clitics retained metrical prominence unlike the case in K’iche’. The verb complex in Mam contains up to four prosodic words (i.e., the markers for aspect, absolutive person, directional clitic, and the main verb), which sets the stage for the expanded use of preverbal directional clitics in Mam and removed the need for the post-verbal clitics except in the case of imperative verbs.

This background informs the comparative approach to the acquisition of K’iche’ and Mam. Pye (1980, 1983) established that phrasal stress was the primary determinant of morpheme production by K’iche’ children. As the examples in (12) show, K’iche’ children frequently omit morphemes that occur before the verb root and sometimes even omit the root itself, but produce morphemes that occur in the stressed position after the verb root. All of the morphemes are obligatory, which rules out input frequency as a determinant of morpheme use. The status suffixes on verbs are portmanteaux morphemes that express aspect, mood, transitivity and derivational status, and are therefore grammatically complex. Nevertheless, the status suffixes are among the first morphemes that K’iche’ children produce.


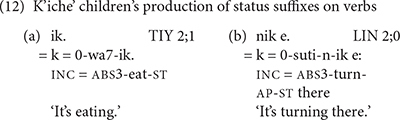


Based on the children’s production of verb affixes in K’iche’, we predict that prosody will guide children’s use of directional clitics in both languages. In K’iche’, because post-verbal clitics are stressed and preverbal clitics are not, we predict that the children will produce the post-verbal directional clitics before they produce the preverbal clitics. In Mam, where the preverbal clitics preserve their status as independent phonological words, we predict that the children will produce both the preverbal and post-verbal directional clitics. The preverbal directional clitics in Mam function as independent phonological words and resist vowel reduction. The post-verbal clitics in Mam follow the stressed syllable in the verb root.

We base these predictions solely on the differences in prosodic structure in K’iche’ and Mam. Because input frequency is considered to be an important factor that directs children’s morphological development, we will pay special attention to disentangling the effects of prosody and input frequency. As we will see in the next section, the use of directional clitics is more frequent in Mam than in K’iche’. The use of post-verbal directional clitics is more frequent than the use of the preverbal directional clitics in K’iche’. The directional clitics in Mam have two metrical advantages over their counterparts in K’iche’: (1) they may be stressed, and (2) they follow the stressed verb syllable in imperative verbs. We analyze the effects of such micro-variation on language acquisition. Because K’iche’ and Mam have similar phonologies, morphologies, syntax and discourse structures, not to mention common patterns of child rearing, we can be more confident in identifying the linguistic sources that account for the differences in the acquisition of directional clitics in K’iche’ and Mam. Table 3 contrasts the predictions from prosody and input frequency for the acquisition of the directional clitics in K’iche’ and Mam.

**TABLE 3:** Contrasting predictions based on prosody and input frequency for the acquisition of directional clitics in K’iche’ and Mam.

	**Preverbal position**	**Post-verbal position**
**Prosody**		

**K’iche’**		
Directional clitics	Late = aspect markers	Early = verb suffixes
Ergative person markers	Late = aspect markers	
**Mam**		
Directional clitics	Early = aspect markers	Early = imperative verb suffix
Ergative person markers	Later than directionals	
Processive clitic		Early = verb suffixes

**Input frequency**		

**K’iche’**		
Directional clitics	Later than aspect markers	Later than verb suffixes
Ergative person markers	Early = aspect markers	
**Mam**		
Directional clitics	Early = aspect markers	Early = imperative verb suffix
Ergative person markers	Early = aspect markers	
Processive clitic		Later than verb suffixes

## Subjects and Methods

We followed the same procedures in recording the Mam and K’iche’ families. Pye made longitudinal audio recordings of the four K’iche’ children in Zunil, Guatemala between 1977 and 1978 (Pye, 1980, 1991); he made longitudinal audio and video recordings of the three Mam children in San Ildefonso Ixtahuacán, Guatemala between 2005 and 2007 (Pye, 2017). Oral and informed consent was obtained from the parents of all participants in K’iche’ and Mam following protocols approved by the human subjects committee of The University of Kansas. The recordings took place in and around the children’s homes. Participants included the children, various members of their families, the K’iche’ and Mam investigators, and visitors to the home. The mothers and siblings were generally present during the recordings, while the fathers only participated occasionally. The families live in rural villages, and the children spend most of their day within the family compound. The K’iche’ and Mam investigators were native speakers of each language and interacted with the children to different degrees. The sessions included play with toys, sticks, plants and picture books.

The data were transcribed in the field by the K’iche’ and Mam investigators; transcripts were annotated by the investigators with contextual and cultural notes. The K’iche’ and Mam investigators added their interpretation of the children’s utterances based on their knowledge of the adult language and culture, the children’s developing phonology and grammar, and the discourse contexts. The transcriptions for both languages were made from the audio recordings.

We selected blocks of two to three 1-h sessions recorded at different ages for each child. Table 4 shows the session numbers and corresponding ages for the K’iche’ children, and Table 5 shows the same information for the Mam children. The analyses of adult speech were made of 1-h recordings of two adults speaking to the K’iche’ child TIY in her first session and two adults speaking to the Mam child WEN in her first session.

**TABLE 4:** Ages, number of utterances, and number and percent of verbs for K’iche’ speakers.

			**Intransitive verbs**		**Transitive verbs**		
		**Number of**					**Percent**
**Speaker**	**Age**	**utterances**	**Types**	**Tokens**	**Types**	**Tokens**	**verbal**
Mother	Adult	382	18	116	27	161	72.5
Adult2 (male)	Adult	211	14	62	23	101	77.3
TIY 1	2;1.17	732	9	24	11	23	6.4
TIY 2	2;7.21	844	33	85	22	117	23.9
TIY 3	2;10.5	1026	34	164	33	130	28.7
LIN	2;0	501	15	50	17	46	19.2
CHA 1	2;9.8	945	18	44	26	178	23.5
CHA 2	3;0.16	1197	43	126	48	232	29.9
CAR	3;1.25	963	30	107	30	140	25.6

**TABLE 5:** Ages, number of utterances, and number and percent of verbs for Mam speakers.

			**Intransitive verbs**		**Transitive verbs**		
		**Number of**					**Percent**
**Speaker**	**Age**	**utterances**	**Types**	**Tokens**	**Types**	**Tokens**	**verbal**
Mother	adult	770	31	125	29	190	40.9
Adult2 (female)	adult	113	9	36	16	32	60.2
WEN 1	1;9.2	1300	41	47	17	44	7.0
WEN 2	2;0.25	3023	31	203	27	399	19.9
WEN 3	2;6	1483	14	107	13	94	13.6
CRU 1	2;5.26	1665	22	87	14	175	15.7
CRU 2	2;11.20	3296	31	318	39	452	23.4
JOS 1	2;7	2213	49	288	25	251	24.4
JOS 2	2;11.10	3298	54	508	40	435	28.6

Table 4 provides the general language measures for the K’iche’ language samples and Table 5 provides the general language measures for the Mam language samples. The last column in each table shows the percentage of the total utterances for each speaker that contained verbs. We excluded the evidential verbs (‘say’) and the existential verbs (‘be somewhere’) from the intransitive verb counts because they are idiomatic expressions and are not inflected for aspect and absolutive agreement.

The number of verb types and tokens are similar across the K’iche’ and Mam speakers. The adults produced a greater percentage of their utterances with verbs than the children. The children produced more exclamations and demonstrative utterances than the adults (Pye, 2017). The older children in the study produced a higher percentage of verbal utterances than the younger children, but they do not approach the frequency of verbal utterances produced by the adult speakers.

## The Production of Directional Verbs in K’iche’ and Mam

We analyzed the child and adult use of the 12 directional verbs shown above in (2) in order to assess the children’s ability to express the direction of motion and their frequency. Examples of the K’iche’ children’s use of intransitive directional verbs appear in (13).


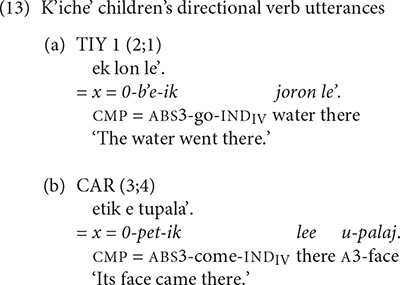


Table 6 shows the number of tokens that each K’iche’ speaker produced for the directional verbs and the percentage of their intransitive verbs that were directional verbs. The children produced a similar number of directional verb types and tokens as the adults. The directional verbs *b’ee* ‘go,’ *peet* ‘come,’ *el* ‘go out,’ *ok* ‘go in’ and *k’is* ‘finish’ were produced by most of the K’iche’ speakers. The directional verbs constitute a relatively large percentage of the younger children’s intransitive verb production. Older children and adults produce a wider range of intransitive verbs.

**TABLE 6:** Directional verb token frequency in K’iche’.

**Directional verb**	**Mother**	**Adult2**	**TIY1 2;1**	**TIY2 2;7**	**TIY3 2;10**	**LIN 2;0**	**CHA1 2;9**	**CHA2 3;0**	**CAR 3;1**
‘go’	11	6	8	22	16	15	13	34	21
‘come’	25	2		5	7	8	2	13	5
‘arrive here’		1		1	1				
‘arrive there’									
‘go down’					4			2	
‘go up’			1	1				2	
‘go out’	6		1	3	15	1	2	3	6
‘go in’	1			7	1	1		3	
‘remain’									
‘return’									
‘pass by’									
‘finish’				8	2	2	2	1	2
Total use	43	9	10	47	46	27	19	58	34
Percent of intransitive verbs	37	14.5	41.7	55.3	28	54	43.2	46	31.8

The Mam speakers produced a wider variety of directional verbs than the K’iche’ speakers. Examples of the Mam children’s use of directional verbs appear in (14). The example in (14c) includes an example with the directional clitic *xi’* ‘go.’


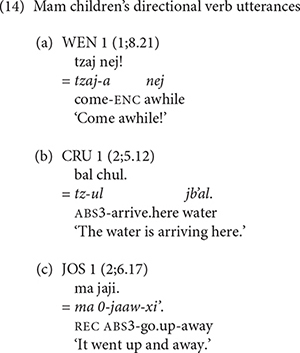


Table 7 shows the number of tokens that each Mam speaker produced for each directional verb. The children generally produced similar numbers of directional verb types and tokens as the adults. The directional verbs *tzaaj* ‘come,’ *xi’* ‘go,’ *kub’* ‘go down,’ *eel* ‘go out,’ *ook* ‘go in’ and *aaj* ‘return’ were produced by most of the Mam speakers.

**TABLE 7:** Directional verb token frequency in Mam.

**Directional verb**	**Mother**	**Adult2**	**WEN1 1;9**	**WEN2 2;0**	**WEN3 2;6**	**CRU1 2;5**	**CRU2 2;6**	**JOS1 2;7**	**JOS2 2;11**
‘go’	35	20	20	23	40	18	28	29	59
‘come’	12	1	4	32	8		46	16	6
‘arrive here’	3		1			4	2	9	3
‘arrive there’					3		2	4	6
‘go down’	1	4	4	6	5	10	18	24	55
‘go up’	2			1	3	3	9	47	110
‘go out’	7	2	3	3	1	6	18	14	22
‘go in’	6	2		4	3	1	3	9	13
‘remain’	4							2	2
‘return’	1		1	6	7		10	1	4
‘pass by’			1	1		1	3		
‘finish’		2		1			14	11	8
Total use	71	31	34	77	70	43	153	166	288
Percent of intransitive verbs	56.8	86	72.3	37.9	65.4	49.4	48.1	57.6	56.7

One analytical problem we had to confront was the frequent use of intransitive directional verbs as substitutes for transitive verbs. We found these substitutions in the speech of both the Mam adults and children. The Mam investigators used the presence of oblique agent phrases, as in (15a and b), to distinguish the omission of a transitive verb in a verb complex with a directional clitic from the use of a directional verb as a substitute for a transitive verb. Examples of the children’s use of intransitive directional verbs as substitutes for transitive verbs appear in (15).


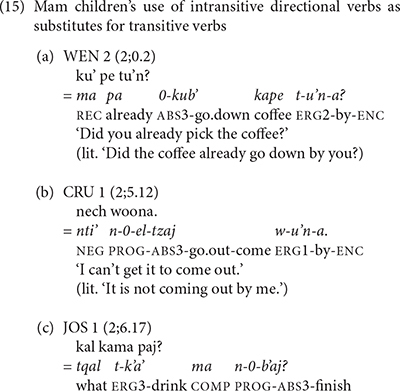



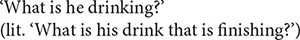


Although the Mam adults produced a greater proportion of directional verbs than the K’iche’ adults, the children produced similar proportions of directional verbs in both languages. The children acquiring Mam produced directional verbs at levels that are similar to the adult levels of production, whereas the children acquiring K’iche’ generally exceeded the adult use of directional verbs. We conclude that the K’iche’ and Mam children have the linguistic ability to express simple motion events early in their language development.

## The Production of Preverbal Directional Clitics in K’iche’ and Mam

There is a marked difference between K’iche’ and Mam speakers in their use of directional clitics. Adult K’iche’ speakers rarely produce verb complexes with preverbal directional clitics. TIY’s mother produced preverbal directional clitics with 5 of 49 verb types. The verb complexes with directional clitics constitute 5.6% of her total verb production. The second K’iche’ adult produced preverbal directional clitics with 1 of 48 verb types, or 1.1% of his total verb production. TIY’s mother produced directional clitics most often with imperative forms of the verb ‘look at.’ The second K’iche’ adult produced a directional clitic with just the verb *chap* ‘catch’ (16) His production of the directional clitic comprised half of his uses of this verb. His use of the preverbal directional clitic was in imperative form. The data indicate that verb complexes with preverbal directional clitics are rare in K’iche’ speech to children.


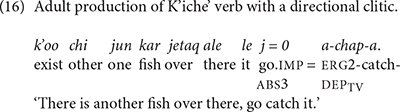


The two youngest K’iche’ children, TIY and LIN, did not produce verb complexes with preverbal directional clitics. TIY did not produce any utterances with interpretations that included preverbal directional clitics. LIN and CHA produced several utterances with preverbal directional clitic interpretations, but did not produce the actual directional clitics. Their examples in (17) contain the dependent suffix that motivates the directional clitic interpretation, but omit the preverbal directional clitic morpheme /–ee/ ‘go.’


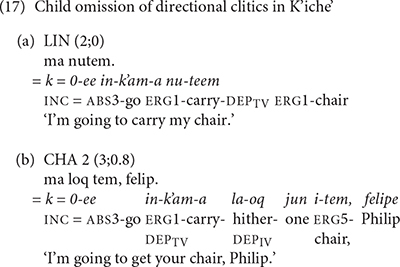


The K’iche’ children in our study begin producing verb complexes with preverbal directional clitics around the age of 3 years. CHA1 (2;9.8) produced an utterance with the suppletive /j-/ form of a preverbal directional clitic in an imperative verb complex (18a). The verb *b’i* ‘say’ is a derived transitive verb that has the status suffix /-ij/ in place of the dependent status suffix that occurs on root transitive verbs with directional clitics. The oldest K’iche’ child, CAR (3;2), produced several verb complexes with preverbal directional clitics. His example in (18b) contains the directional clitic *ul* ‘come’ and his example in (18c) contains the suppletive /j-/ form of directional clitic in an imperative verb complex.


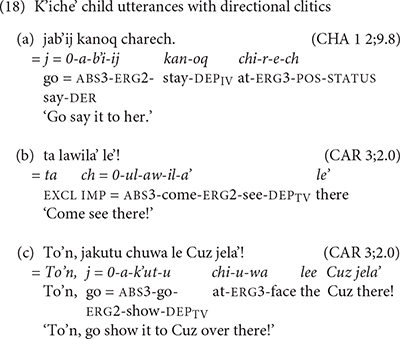


The number of times the K’iche’ speakers produced verbs with preverbal directional clitics are shown in Table 8. Percentages for verb types with directional clitics are proportional to the total number of verb types used by each speaker. Percentages for verb tokens with directional clitics are proportional to the number of tokens of the verb types used at least once with directionals. The 6 tokens with directional clitics that CAR produced constitute 17.1% of his use of the verbs he produced with directionals, but only constitute 2.4% of his total verb production.

**TABLE 8:** Child and adult production of verb complexes with preverbal directional clitics in K’iche’.

	**Verb types**		**Verb tokens**	
		
	**Number**	**Percent**	**Number**	**Percent**
TIY’s mother	5	10.2	15	5.6
Adult2	1	2.1	1	1.2
TIY1 2;1		0		0
TIY2 2;7		0		0
TIY3 2;10		0		0
LIN 2;0		0		0
CHA1 2;9	1	2.1	1	0.3
CHA2 3;0		0		0
CAR 3;1	3	5	6	17.1

In contrast with the rarity of the production of preverbal directional clitics in K’iche’, Mam adults made frequent use of preverbal directional clitics in their speech to children. WEN’s mother’s production of verb complexes with directional clitics constituted 23% of her verbal utterances. The second Mam adult’s use of verb complexes with directional clitics constituted 31% of her verbal utterances. WEN’s mother used preverbal directional clitics with 20 of 54 different verb types. The second Mam adult used directional clitics with 13 of 22 different verb types.

The children acquiring Mam produced verb complexes with preverbal directional clitics in their earliest recordings. WEN’s example in (19a) shows the use of the directional clitic *tzaj* ‘hither’ contracted to /tz/ in post-verbal position. CRU’s example in (19b) shows the use of the directional clitic *jaw* ‘up’ preceding the transitive verb *q’i* ‘carry’ with the dependent suffix -’*n* required for transitive verbs with directional clitics. JOS’s example in (19c) contains the directional clitic *kub’* ‘down’ preceding the intransitive verb *tan* ‘sleep.’


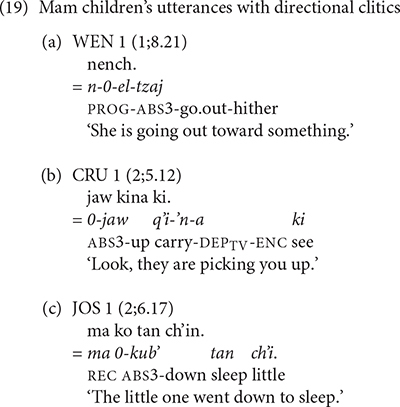


England (1983, p. 169) notes that the directional clitics shift to a position following the main verb in two contexts: (1) when the main verb is a directional verb, or (2) when the main verb is in the imperative mood [see example (5b) above]. The children’s mastery of these shifts is especially impressive as shown in the following examples. In (20a) WEN tells the investigator to give something using an imperative verb with the directional clitic *tzaj* ‘come.’ In (20b) CRU comments that something came down using the directional verb *kub’* ‘go down’ and the directional clitic *tzaj* ‘hither.’ In (20c) JOS issued a demand using the directional clitic *xi* ‘away.’ We note that the children use the same contracted forms of the directional clitics that adults use when the directional clitics follow the main verb.


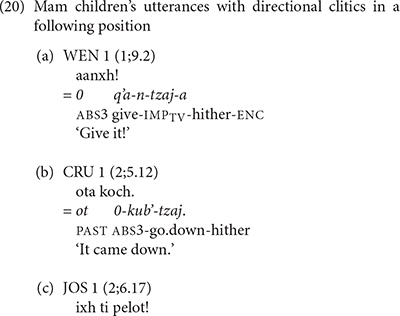



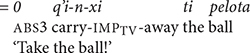


We also find evidence that the Mam children produced sequences of directional clitics with the verbs. WEN produced contrasting verb forms that contained one and two directional clitics. In (21), WEN used contrasting directional clitics with the verb *q’i* ‘carry.’ In (21a) WEN used the verb with the contracted form of the directional -*xi* ‘away.’ In (21b) she produced the same verb with the interpretation that has contracted forms of the directional clitics -*aj* ‘return’ and -*tzaj* ‘hither.’ In (22), JOS produced the verb *tz’aq* ‘fall’ with contracted forms of the directional clitics *el* ‘out’ and *xi* ‘away.’


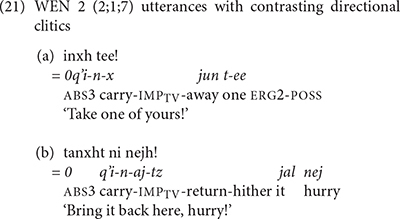



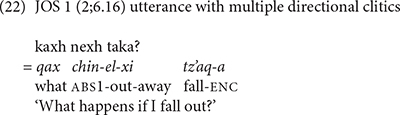


The quantitative results for the Mam speakers are shown in Table 9. Once again, the percentages for verb types with directional clitics relate to the total number of verb types used by each speaker. Percentages for verb tokens with directional clitics are relative to the number of tokens of the verb types used at least once with directionals.

**TABLE 9:** Child and adult production of verb complexes with directional clitics in Mam.

	**Verb types**		**Verb tokens**	
		
	**Number**	**Percent**	**Number**	**Percent**
WEN’s mother	20	37	107	22.9
Adult2	13	59	21	30.9
WEN1 1;9	8	25.8	17	17.9
WEN2 2;0	11	19	48	12.4
WEN3 2;6	10	37	24	33.3
CRU1 2;5	16	31.3	31	12.2
CRU2 2;11	29	41.4	97	40.8
JOS1 2;7	44	59.5	223	62.1
JOS2 2;11	41	43.6	339	51.1

Although the K’iche’ children in the study could express direction by means of the intransitive verbs, they did not produce verb complexes with preverbal directional clitics until around the age of 2 years and 9 months. The initial production of preverbal directional clitics comes in the context of imperative verbs with the suppletive form of the verb *b’ee* ‘go.’ The children’s ability to express direction by means of intransitive verbs shows that their inability to produce verb complexes with preverbal directional clitics is not due to a lack of knowledge of direction or directional verbs.

The story is very different for the children acquiring Mam in the study. Not only did they produce verb complexes with preverbal directional clitics early, they did so with a variety of verbs in declarative and imperative moods. Our youngest Mam children were already producing instances of directional clitics in their first recordings. The early production of preverbal directional clitics in Mam is evidence that the grammatical complexity of verb complexes with directional clitics does not explain their omission in the speech of the K’iche’ children. We can, therefore, rule out cognitive constraints as a determining factor in the children’s production of preverbal directional clitics.

The rarity of verb complexes with preverbal directional clitics in K’iche’ might lead to a sampling error in that it might be necessary to record K’iche’ children for longer periods of time in order to record the use of rare constructions like directional clitics. The children’s examples in (17) are telling in this regard in that they show utterances that omit prefixes in the verb complex, but contain the dependent verb suffix that is evidence for the intention to use directional clitics. We conclude that while the production of directional clitics is relatively late in the K’iche’ children’s speech, they display an early awareness of the grammar of verb complexes with directional clitics. The children’s use of the obligatory dependent verb suffixes in contexts of verbs with directional clitics in both languages is especially impressive.

## The Production of Post-Verbal Directional Clitics in K’iche’

Adult K’iche’ speakers produce more verbs with post-verbal directional clitics than verb complexes with preverbal directional clitics. The second K’iche’ adult produced post-verbal directional clitics with nine verbs (*b’an* ‘do,’ *chap* ‘grab,’ *eqaj* ‘carry on back,’ *il* ‘see,’ *jururej* ‘drag,’ *k’am* ‘carry in arms,’ *k’ol* ‘guard,’ *tzijon* ‘chat,’ *ya’* ‘put’) in a 1-h session in which he produced a total of 13 intransitive verbs and 23 transitive verbs. The majority of uses are with verbs of physical transfer (*eqaj* ‘carry on back,’ *jururej* ‘drag,’ *k’am* ‘carry in arms, *ya’* ‘put’). Nine of his 20 post-verbal directional clitics occurred with the verb *k’am* ‘carry’ (23). He produced utterances with the verb *k’am* 15 times. We will use the adult production of post-verbal directional clitics as a baseline for evaluating the children’s use of the post-verbal directional clitics.


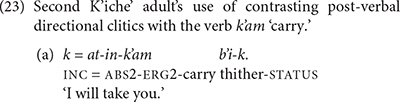



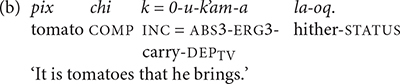


The K’iche’ children also produced verbs with post-verbal directional clitics, but their use was more restricted than the adults’. TIY only produced the directional *la*-*oq* ‘hither’ twice with the verb *k’am* ‘carry.’ She did not produce the verb, which we inferred from its context of use (24a). The 2-year-old boy LIN also omitted the verb in most of his utterances with directional particles. He produced the verb root in the example shown in (24b). LIN only produced directional particles with the verbs *k’am* ‘carry’ and *ya’* ‘give, put.’ As they grew older, the K’iche’ children expanded the number of verbs that they produced with directional particles. Past two and a half years, TIY produced directional particles with five different verb types and CHA produced directional particles with 7 different verb types. One of CHA’s utterances in shown in (24c). All of the children’s productions contain the phrase-final form of the directional clitic containing a suffix.


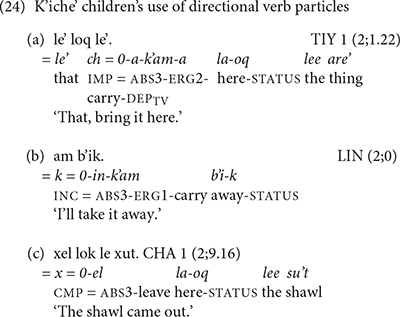


Because of the association between verbs and post-verbal directional clitics, we counted the number of times the speakers produced verbs with directional clitics as well as the number of times that the speakers produced the same verbs without post-verbal directional clitics to assess the child and adult production of post-verbal directional clitics. We then calculated the percent use of directional tokens for those verbs that speakers produced with post-verbal directional clitics. The results are shown in Table 10.

**TABLE 10:** Production of post-verbal directional clitics in K’iche’.

	**Directional**		**Verbs used**		
	**clitic use**		**with directionals**		
					**Percent**
**Adult**	**Types**	**Tokens**	**Types**	**Tokens**	**tokens**
Mother – TIY	4	49	14	98	50
Adult2	3	19	8	57	33
TIY1 2;1	1	2	1	3	67
TIY2 2;7	3	12	5	100	12
TIY3 2;10	5	20	8	80	25
LIN 2;0	2	7	2	14	50
CHA1 2;9	3	13	7	73	18
CHA2 2;10	2	18	7	50	36
CAR 3;1	2	4	2	36	11

The results suggest that K’iche’ children may produce more instances of directional clitics as they get older, though this pattern only holds strongly for TIY, and the oldest child (CAR) only produced four utterances with post-verbal directional clitics. The 2-year-old children produced post-verbal directional clitics even if they did not produce the verb together with the directional clitics. The adult speakers produced post-verbal directional clitics with a greater number of verb types than the children, but the children produced post-verbal directional clitics with similar percentages of verb tokens as adults. We conclude that the 2-year-old K’iche’ children used a limited number of post-verbal directional clitics (primarily the directional *la*-*oq* ‘hither’) in association with a limited number of verbs (primarily the verbs *k’am* ‘carry’ and *ya’* ‘give, put’). Between 2 and 3-years-of-age they produced more types of post-verbal directional clitics with more types of verbs. The children’s production of post-verbal directional clitics appeared to precede their production of preverbal directional clitics by several months.

## The Production of the Processive Suffix in Mam

We also found evidence that Mam children produced instances of the processive verb suffix early in their development. Examples of the children’s processive suffix use are shown in (25). As these examples show, we only found examples of children using the imperative form of the processive suffix. In (25a), WEN produced the imperative processive suffix with the verb *il* ‘see.’ This is one of the three Mam verbs that is not used with directional clitics. Most of children’s examples were used with this verb. The example in (25c) shows the use of the processive suffix with the verb *q’ii* ‘carry.’ Both the verbs *il* ‘see’ and *q’ii* ‘carry’ are frequent in the speech of Mam adults and children, but speakers seldom add the processive suffix. The second adult speaker for Mam and the child CRU did not produce any tokens of the processive suffixes in their samples. Table 11 shows the number of verbs that the Mam participants produced with the processive suffix as well as the percentage of the tokens of these verbs that had the suffix.


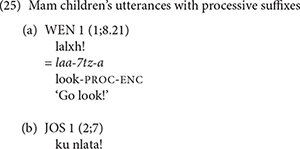



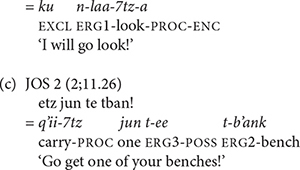


**TABLE 11:** Processive suffix use in Mam.

		**Verb use**		
	**Processive**			**Percent**
**Adult**	**use tokens**	**Types**	**Tokens**	**tokens**
Mother – WEN	4	3	137	2.9
Adult2				No data
WEN 1 1;9	4	3	23	17.3
WEN 2 2;0	1	1	4	25
WEN 3 2;6				No data
CRU 1 2;5				No data
CRU 2 2;11				No data
JOS 1 2;7	2	1	12	16.7
JOS 2 2;11	6	2	30	20

## Comparative Analysis of Directional Acquisition in K’iche’ and Mam

K’iche’ and Mam add directional clitics to movement and transfer verbs in order to specify the agent’s direction of motion. The languages differ in that K’iche’ favors the use of post-verbal directional clitics, while Mam favors the use of preverbal directional clitics. K’iche’ only has three preverbal directional clitics, whereas Mam has 12 preverbal directional clitics. The difference in the number of preverbal directional clitics in K’iche’ and Mam is associated with a marked difference in their prosodic features. The preverbal clitics in K’iche’ are unstressed, whereas the preverbal clitics in Mam may be stressed, and do not undergo unstressed vowel deletion. The directional clitics in Mam move to a post-verbal position on imperative verbs due to the absence of a host morpheme. The post-verbal clitics in K’iche’ have a status suffix that appears when the post-verbal clitics have phrase-final stress.

Pye (1980, 1983) established that phrasal stress was the primary determinant of morpheme production by K’iche’ children. K’iche’ children frequently omit morphemes that occur before the verb root, but produce morphemes that occur in the stressed position after the verb root. Based on the children’s production of verb affixes in K’iche’, we predicted that children acquiring K’iche’ and Mam would produce directional clitics that have lexical stress, or that follow the verb. For K’iche’, we predicted that the children would produce the post-verbal directional clitics before they produced the preverbal clitics. For Mam, we predicted that the children would produce both the preverbal and post-verbal directional clitics as well as the processive verb suffix.

The results reported in the previous section support these predictions. Only the oldest K’iche’ children, CHA and CAR, produced any preverbal directional clitics, whereas, even the youngest Mam children produced preverbal directional clitics. The Mam children produced post-verbal directional clitics on imperative verbs and intransitive, directional verbs in accordance with the adult grammar. All of the K’iche’ children produced post-verbal directional clitics. We also found evidence that the K’iche’ and Mam children follow the prosodic and grammatical constraints on the use of directional clitics in their languages. The K’iche’ children added the status suffixes to post-verbal clitics in phrase-final position. The Mam children added the dependent status morpheme to transitive verbs that they produced with preverbal directional clitics. The Mam children also observed the constraints on unstressed vowels in their combined forms of directional clitics in both the preverbal and post-verbal positions. The forms and frequencies of directional clitics serve as distinctive grammatical markers of children’s speech in K’iche’ and Mam.

The children’s production of directional clitics could be tied to their frequency in adult speech. The two K’iche’ adults only produce preverbal directional clitics with 2 to 10% of verb types and 1 to 6% of verb tokens. The two Mam adults produce preverbal directional clitics with 37 to 59% of verb types and 22 to 30% of verb tokens. There is also a qualitative difference between K’iche’ and Mam in the use of directional clitics. Mam has 12 preverbal directional clitics, whereas K’iche’ only has three preverbal directional clitics. Mam allows for sequences of up to three preverbal directional clitics, whereas K’iche’ only licenses the use of a single directional clitic in its verb complex.

The grammatical structures of K’iche’ and Mam allow us to tease apart the effects of frequency and prosody by examining the acquisition of high frequency, unstressed morphemes and low frequency, stressed morphemes. The preconsonantal ergative person markers in Table 1 provide ideal examples of high frequency, unstressed morphemes in K’iche’ and Mam in that K’iche’ and Mam have similar sets of ergative person markers and place them in the same position in the verb complex. The children omit the unstressed ergative agreement markers in both languages even though these inflections are obligatory in adult speech. K’iche’ 2-year-old children produce preconsonantal ergative agreement markers in less than 20% of their obligatory contexts; Mam 2-year-olds produce preconsonantal ergative markers in a third of their obligatory contexts (Pye, 2017, p. 190).

We can also examine the acquisition of stressed, low frequency morphemes in K’iche’ and Mam. As shown above in Table 10, the post-verbal directional clitics are somewhat infrequent in K’iche’ in that they occur with half of the verb tokens in the mother’s speech and in a third of the verb tokens in the investigator’s speech. While the post-verbal directional clitics are not as rare as the preverbal directional clitics in adult speech, they are by no means ubiquitous in K’iche’ speech to children. The directional particles can receive the primary stress for the verb phrase, and we found evidence that 2-year-old K’iche’ children produce the post-verbal directional clitics in their speech.

We also showed that the processive suffixes are rarely produced in speech to Mam children. Table 11 shows that the mother produced the processive suffixes in 3% of her verb tokens; the second Mam adult did not produce any processive suffixes in her speech. The processive suffixes follow the stressed syllable in the verb and are therefore as prosodically salient as the directional clitics that follow imperative and directional verbs. Despite their low input frequency we find evidence of their use in the early speech of Mam children.

Based on these results from an in-depth, multi-layered investigation of these seven children’s early speech patterns, we propose that while a high input frequency can help children notice some inflectional features, prosody plays a primary role in determining which parts of a verb complex children produce. Two-year-old children acquiring K’iche’ respond to the structural features of the language by producing the syllables in the verb complex with primary stress and thereby produce verb suffixes and post-verbal directional clitics. Two-year-old children acquiring Mam respond to its structural features by producing the metrically prominent syllables in the Mam verb complex and thereby produce both preverbal and post-verbal directional clitics. Their use of directional clitics in preverbal and post-verbal positions within the indicative and imperative verb complexes as well as with the intransitive directional verbs is especially striking.

## Conclusion

We used the comparative method to identify the main factors at play in children’s production of directional clitics in K’iche’ and Mam. K’iche’ and Mam inherited an Eastern Mayan verbal complex with markers for aspect, object, subject and verb status. Both languages add directional clitics to their verb template to specify the path of the agent. We argue that over the course of the past 3000 years, the prosodic structure of the verb complex took different paths in the Eastern Mayan languages. K’iche’ shifted its primary lexical stress to the final syllable and added phrasal stress at the end of the verb phrase. Mam weakened vowels in unstressed syllables and developed a rule for adding stress to heavy syllables. The changes to the prosodic structure of the verb complex had dramatic effects on the use of the directional clitics. K’iche’ speakers seldom produce directional clitics in preverbal position, whereas directional clitics have become a hallmark of Mam.

The historical changes to the prosodic structure in K’iche’ and Mam also had consequences for the acquisition of the two languages. We showed that predictions derived from children’s production of inflectional morphemes in K’iche’ could be extended to children’s production of directional clitics in K’iche’ based on their prosodic features. We then extended the same predictions to children’s production of directional clitics in Mam based on their prosodic features. We hypothesize that prosody directs children’s production of verb complexes in the other Eastern Mayan languages as well. Language history determines which parts of the verb complex are prosodically salient.

Pye (1980, 1983) suggested that prosody could account for children’s productions in other languages besides K’iche’. Other researchers have since made similar suggestions to account for children’s productions in various languages (Peters, 1983; Mithun, 1989; Demuth and Fee, 1995; Gerken, 1996; cf. Deen, 2005; Terry, 2010; Forshaw, 2016). It is important to remember that prosody is far from a unitary linguistic feature. It varies across languages as much as word order and morphological complexity (Evans and Levinson, 2009; Stassen, 2011). Even K’iche’ and Mam differ in their deletion of vowels in unstressed syllables. It is therefore necessary to have a better understanding of prosodic realization in many 1000s of languages before claiming that prosody *per se* accounts for children’s productions. This paper makes a start in this direction.

### Orthography

All Mayan words are shown in the practical orthography developed by the Proyecto Lingüístico Francisco Marroquín (Kaufman, 1976). The orthographic symbols have their standard IPA values except: 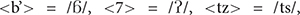 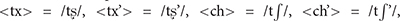 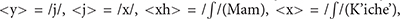 <x>=/s/Mam.

## Data Availability Statement

The datasets generated for this study are available on request to the corresponding author.

## Ethics Statement

The studies involving human participants were reviewed and approved by The University of Kansas human subjects committee. Written informed consent from the participants’ legal guardian/next of kin was not required to participate in this study in accordance with the national legislation and the institutional requirements.

## Author Contributions

CP recorded and analyzed the K’iche’ and Mam data, and wrote the first draft of the manuscript. BP organized the data and edited the manuscript.

## Conflict of Interest

The authors declare that the research was conducted in the absence of any commercial or financial relationships that could be construed as a potential conflict of interest.

## Abbreviations

1: first person singular
2: second person singular
3: third person singular
4: first person plural
5: second person plural
ABS: absolutive person marker
AP: antipassive
CMP: completive aspect
COMP: complementizer
DEP: dependent status suffix
DER: derived verb status suffix
ENC: enclitic person marker
ERG: ergative person marker
exc: exclusive
EXCL: exclamation
FAM: familiar marker
IMP: imperative
INC: incompletive aspect
IND: indicative status suffix
iv: intransitive verb
ixc: inclusive
NEG: negative
PAST: remote past tense
pl: plural
POS: possessive
PROC: processive
PROG: progressive aspect
REC: recent past tense
sg: singular
STATUS: status suffix
tv: transitive verb.
